# Wearable Health-Tracking Devices and Adherence to Physical Activity Guidelines Among US Adults, Including Those with Elevated Anxiety Symptoms: A Nationally Representative Cross-Sectional Analysis of HINTS 7 (2024)

**DOI:** 10.1177/15598276261496228

**Published:** 2026-10-10

**Authors:** David Goldman, Laurie Kortowich, Matthew Nagra

**Affiliations:** 1 Department of Public Health, 3835 University of Helsinki, Helsinki, Finland (DG); 2 Department of Research and Development, Metabite, Inc., New York, NY, USA (DG); 3 Department of Health and Innovation, Plant Strong, Austin, TX, USA (LK); 4 Department of Family Practice, University of British Columbia, Vancouver, Canada (MN)

**Keywords:** lifestyle medicine, physical activity, wearable devices, self-monitoring, anxiety, digital health, health equity, HINTS

## Abstract

Physical activity is a pillar of lifestyle medicine, yet most US adults do not meet aerobic guidelines, and inactivity is especially common among people with anxiety. Wearables enable self-monitoring, but population-level evidence linking them to guideline adherence among distressed adults is limited. We analyzed 6686 adults from the nationally representative Health Information National Trends Survey 7 (2024). The outcome was meeting the aerobic guideline (≥150 weekly minutes of at least moderate-intensity activity); the exposure was wearable device use for health tracking. Elevated anxiety was defined as a 2-item Generalized Anxiety Disorder scale score ≥3. Weighted logistic regression yielded odds ratios (ORs) adjusted for sociodemographic and health covariates. Wearable users were more likely than non-users to meet the guideline (48.1% vs 36.4%; adjusted OR, 1.59; 95% CI, 1.33-1.91; *P* < .001). The association was consistent across anxiety strata (interaction *P* = .33); among distressed adults, 48.2% of users vs 28.1% of non-users met the guideline. In secondary analyses, wearable use was not associated with meeting ≥300 min/wk (adjusted OR, 1.09; 95% CI, 0.84-1.40). Wearable use was independently associated with meeting activity guidelines, including among adults with elevated anxiety, supporting its potential as a scalable lifestyle-medicine adjunct. Cross-sectional data preclude causal inference.

“Adults with anxiety and depression are markedly less active than the general population and face distinctive motivational barriers.”

## Introduction

Regular physical activity is one of the six pillars of lifestyle medicine and among the most powerful modifiable determinants of chronic-disease risk and premature mortality.^1,2^ The 2018 Physical Activity Guidelines for Americans recommend that adults accumulate at least 150 minutes of moderate-intensity aerobic activity per week, yet nearly 80% of US adults fail to meet the combined guidelines for both aerobic and muscle-strengthening activity, sustaining what has been described as a global pandemic of physical inactivity.^3-5^ In 2019, the Global Burden of Disease study estimated that 15.74 million disability-adjusted life years were lost and 0.83 million deaths occurred globally due to low physical activity.^6^ Closing the gap between physical activity guidelines and current physical activity levels is a central objective of both clinical lifestyle medicine and population health.

The burden of inactivity is unevenly distributed. Adults with common mental-health conditions, particularly anxiety and depression, engage in substantially less moderate-to-vigorous activity and more sedentary time than the general population, despite robust evidence that physical activity both prevents and reduces depressive and anxiety symptoms.^7-12^ This is a clinically consequential paradox: the population that stands to gain the most from activity often faces the greatest motivational and structural barriers to achieving it.^13^

Wearable health-tracking devices, including consumer smartwatches and fitness trackers, have been proposed as a scalable tool to help close this gap. Their core mechanism, continuous self-monitoring paired with feedback and goal-setting, maps directly onto control theory and social cognitive theory, and reflects the behavior-change techniques most consistently associated with increased physical activity.^14-17^ By making activity visible, quantifiable, and goal-referenced, wearables can support the autonomous motivation and self-regulation that sustain behavior over time. Randomized and pooled evidence indicate that wearable trackers produce modest but reliable increases in physical activity across diverse populations, with some trials also reporting improvements in depressive symptoms, although this evidence remains limited and inconsistent.^18-22^

However, most of this evidence derives from selected trial samples, and comparatively little is known about whether device use is associated with physical activity guideline adherence at the population level, or whether any such association extends to adults with elevated psychological distress, precisely the group for whom self-regulatory support may be most valuable. Understanding these associations has direct implications for lifestyle-medicine practice and for digital-health equity. If wearable use is associated with meaningfully higher guideline adherence, and if that association holds among psychologically distressed adults, clinicians and public-health programs would have empirical grounds to consider self-monitoring technology as a broadly applicable behavioral adjunct.

Using the newly released, nationally representative Health Information National Trends Survey 7 (HINTS 7, 2024), we (1) estimated the association between wearable device use and meeting aerobic activity guidelines among US adults, adjusting for sociodemographic and health covariates; and (2) tested whether this association is modified by elevated anxiety symptoms. We hypothesized that wearable use would be independently associated with higher odds of meeting activity guidelines and that the association would be at least as strong among adults reporting elevated anxiety.

## Methods

### Data Source and Sample

We analyzed public-use data from HINTS 7 (2024), a cross-sectional, nationally representative mailed survey of the US noninstitutionalized adult population (aged ≥18 years) administered by the National Cancer Institute.^23,24^ HINTS uses a stratified probability design in which addresses are sampled from a national frame, and one adult per household is selected; the 2024 cycle yielded 7278 completed surveys. From this cycle, we excluded respondents missing data on wearable use or the physical activity items, yielding an analytic sample of 6686 respondents; fully adjusted models used 6046 respondents with complete covariate data. Because the analysis used de-identified, publicly available data, it was exempt from institutional review board review. This study followed the STROBE reporting guideline for cross-sectional studies.

### Outcome: Meeting the Aerobic Activity Guideline

Respondents reported the number of days per week they did any physical activity or exercise of at least moderate intensity (e.g., brisk walking, or bicycling or swimming at a regular pace; excluding weightlifting) and the typical duration of each session, in minutes. Weekly moderate-activity minutes were computed as the product of frequency and duration; respondents reporting zero days were assigned zero minutes. HINTS 7 does not include a separate item on vigorous-intensity activity; vigorous activity is therefore captured within this single item, but is counted minute-for-minute rather than double-weighted, as the Guidelines allow when calculating moderate-equivalent minutes.^3,4^ Consistent with the 2018 Physical Activity Guidelines for Americans,^3^ we defined the primary binary outcome indicating whether a respondent accumulated at least 150 weekly minutes of at least moderate-intensity activity. The Guidelines note that additional health benefits accrue with more than 300 minutes per week,^3,4^ so we also defined 2 secondary high-guideline outcomes: (1) ≥300 vs < 300 weekly minutes and (2) ≥300 vs < 150 weekly minutes, the latter excluding adults reporting 150-299 minutes.

### Exposure: Wearable Device Use

The exposure was derived from the HINTS item asking whether respondents had used an electronic wearable device (e.g., a fitness band or smartwatch) to monitor or track their health during the prior 12 months. Respondents were classified as wearable users vs non-users.

### Effect Modifier: Elevated Anxiety Symptoms

Psychological distress was assessed with items from the Patient Health Questionnaire-4 (PHQ-4), a validated ultra-brief screen combining a 2-item anxiety subscale (Generalized Anxiety Disorder-2 [GAD-2]) and a 2-item depression subscale (PHQ-2).^25-27^ Response options were coded from 0 (not at all) to 3 (nearly every day) so that higher scores denote greater symptom burden. Following established conventions,^25,27^ elevated anxiety symptoms were defined as a GAD-2 score of 3 or greater (range, 0-6). Analyses using the full 4-item PHQ-4 yielded consistent patterns.

### Covariates

Models adjusted for age (years), sex (female vs male), education, household income, body mass index (calculated as weight in kilograms divided by height in meters squared), self-rated general health (higher categories indicating worse health), and race/ethnicity (non-Hispanic White [reference], non-Hispanic Black, Hispanic, non-Hispanic Asian, and other). These covariates were selected a priori as established correlates of both device adoption and physical activity.^5,22^

### Statistical Analysis

All estimates incorporated the HINTS final person-level sampling weight to produce nationally representative results. Although the HINTS public-use file supports both jackknife replication and Taylor-series linearization, standard errors and 95% CIs were computed using the 50 jackknife replicate weights provided (49 denominator degrees of freedom), the method recommended by the National Cancer Institute for public-use data, because replication reflects the variance reduction achieved by raking the survey weights.^28,29^ Weighted logistic regression models estimated ORs for meeting the activity guideline: an unadjusted model, a fully adjusted model, and a model adding an anxiety indicator with a multiplicative wearable × anxiety interaction term. To characterize the association across mental-health status, we additionally fit fully adjusted models stratified by anxiety status. Secondary analyses repeated the unadjusted, fully adjusted, interaction, and stratified models for the 2 high-guideline outcomes. Descriptive statistics were weighted. Two-sided *P* < .05 defined statistical significance; actual *P* values are reported unless less than .001. Analyses were conducted in R (version 4.3.2) using the survey package to incorporate the HINTS jackknife variance estimator.

## Results

### Sample Characteristics

Weighted characteristics of the analytic sample are shown in Table 1. The population had a mean age of 48.8 years and was 48.8% female; 40.9% reported using a wearable device to track health. Wearable users were younger (44.9 vs 51.5 years), more likely to have completed college (43.6% vs 27.5%) and to report higher household income (57.0% vs 36.5%), and less likely to report fair or poor general health (12.6% vs 20.4%) than non-users, a profile consistent with documented sociodemographic gradients in device adoption. Notably, the prevalence of elevated anxiety symptoms was identical among users and non-users, at 17.9% each (Table 1).

**Table 1.** Weighted Characteristics of US Adults in the Analytic Sample, Overall and by Wearable Device Use, HINTS 7 (2024) (N = 6686)

Characteristic	Overall	Wearable Users	Non-users
Unweighted n	6686	2502	4184
Age, mean, y	48.8	44.9	51.5
Female, %	48.8	52.2	46.4
Body mass index, mean, kg/m^2^	28.8	29.2	28.6
College graduate, %	34.1	43.6	27.5
Higher household income, %	44.9	57.0	36.5
Fair/poor general health, %	17.2	12.6	20.4
Non-Hispanic Black, %	10.4	10.5	10.3
Hispanic, %	16.5	16.9	16.2
Non-Hispanic Asian, %	5.4	4.9	5.7
Elevated anxiety symptoms, %	17.9	17.9	17.9
Meets activity guideline, %	41.2	48.1	36.4
Weekly activity <150 min, %	58.8	51.9	63.6
Weekly activity 150-299 min, %	20.9	27.3	16.5
Weekly activity ≥300 min, %	20.2	20.8	19.9

*Note.* Percentages and means are weighted to the US adult population using the HINTS final person weight. “Higher household income” denotes annual household income of $75,000 or more (NCI-imputed income variable); “college graduate” denotes a bachelor’s degree or higher. Weekly activity categories refer to minutes per week of at least moderate-intensity activity. Sex was coded from birth sex; percentages exclude missing values.

### Wearable Use and Meeting Activity Guidelines

Wearable users were substantially more likely than non-users to meet the aerobic activity guideline (48.1% vs 36.4%; Figure 1). In unadjusted weighted logistic regression, wearable use was associated with markedly higher odds of meeting the guideline (OR, 1.62; 95% CI, 1.40-1.88; *P* < .001). This association was only minimally attenuated after full adjustment for age, sex, education, income, body mass index, general health, and race/ethnicity (adjusted OR, 1.59; 95% CI, 1.33-1.91; *P* < .001; Table 2, Figure 2). Among covariates, female sex (OR, 0.67; 95% CI, 0.54-0.82), higher body mass index (OR, 0.98 per unit; 95% CI, 0.96-0.99), and worse self-rated general health (OR, 0.64 per category; 95% CI, 0.58-0.71) were associated with lower odds. Age, education, income, and race/ethnicity were not independently associated with the outcome after adjustment (Table 2).

**Figure 1.**
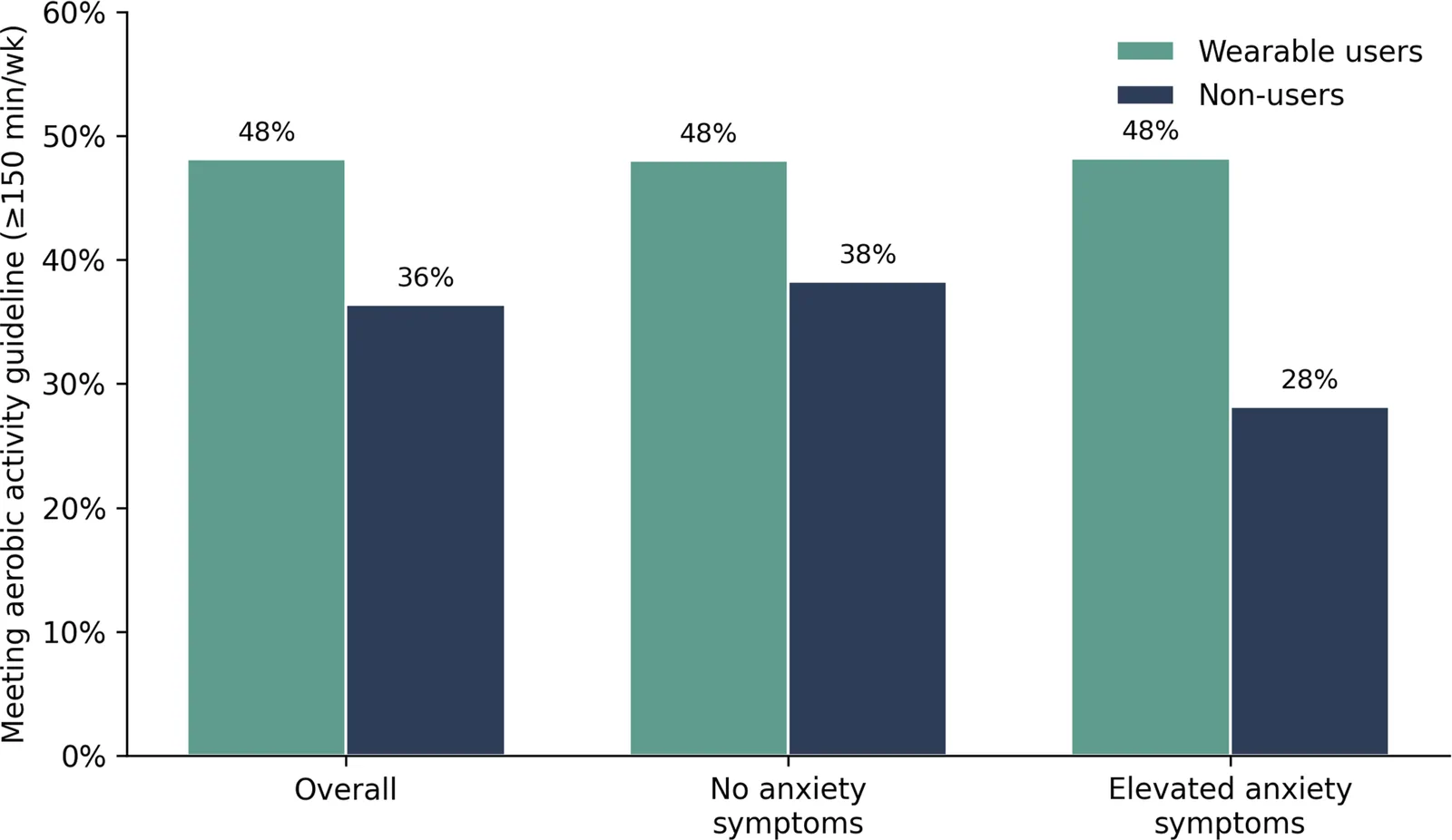
Weighted prevalence of meeting the aerobic activity guideline (≥150 min/week) by wearable device use, overall and stratified by anxiety symptom status, HINTS 7 (2024)

**Table 2.** Weighted Logistic Regression Odds Ratios for Meeting the Aerobic Activity Guideline (≥150 min/wk) and Secondary High-Guideline Outcomes Among US Adults, HINTS 7 (2024)

Predictor	OR	95% CI	*P*
Primary exposure			
Wearable device use (vs none), unadjusted	1.62	1.40-1.88	<.001
Wearable device use (vs none), adjusted	1.59	1.33-1.91	<.001
Covariates (fully adjusted model)			
Female (vs male)	0.67	0.54-0.82	<.001
Age (per year)	1.00	1.00-1.00	.97
Education (per level)	1.00	0.93-1.07	.94
Income (per level)	1.01	0.96-1.07	.65
Body mass index (per unit)	0.98	0.96-0.99	<.001
Worse general health (per level)	0.64	0.58-0.71	<.001
Non-Hispanic Black (vs White)	0.81	0.60-1.08	.15
Hispanic (vs White)	1.17	0.87-1.58	.30
Non-Hispanic Asian (vs White)	0.76	0.47-1.21	.25
Effect modification by anxiety			
Wearable × elevated anxiety (interaction)	1.37	0.73-2.61	.33
Wearable use among distressed (stratified)	1.75	0.91-3.36	.10
Wearable use among non-distressed (stratified)	1.54	1.30-1.83	<.001
Secondary outcomes: Wearable device use (vs none)			
≥300 vs < 300 min/wk, unadjusted	1.06	0.85-1.31	.61
≥300 vs < 300 min/wk, adjusted	1.09	0.84-1.40	.52
≥300 vs < 150 min/wk, unadjusted	1.28	1.04-1.57	.02
≥300 vs < 150 min/wk, adjusted	1.34	1.04-1.71	.02

*Note.* OR indicates odds ratio; CI, confidence interval. Estimates are weighted to the US adult population; 95% CIs and *P* values derive from jackknife replicate-weight variance estimation using 50 replicate weights. Fully adjusted model n = 6046; stratified distressed n = 983; non-distressed n = 5018. Secondary outcome models: ≥300 vs < 300 min/wk, fully adjusted n = 6046; ≥300 vs < 150 min/wk (adults reporting 150-299 min/wk excluded), fully adjusted n = 4762.

**Figure 2.**
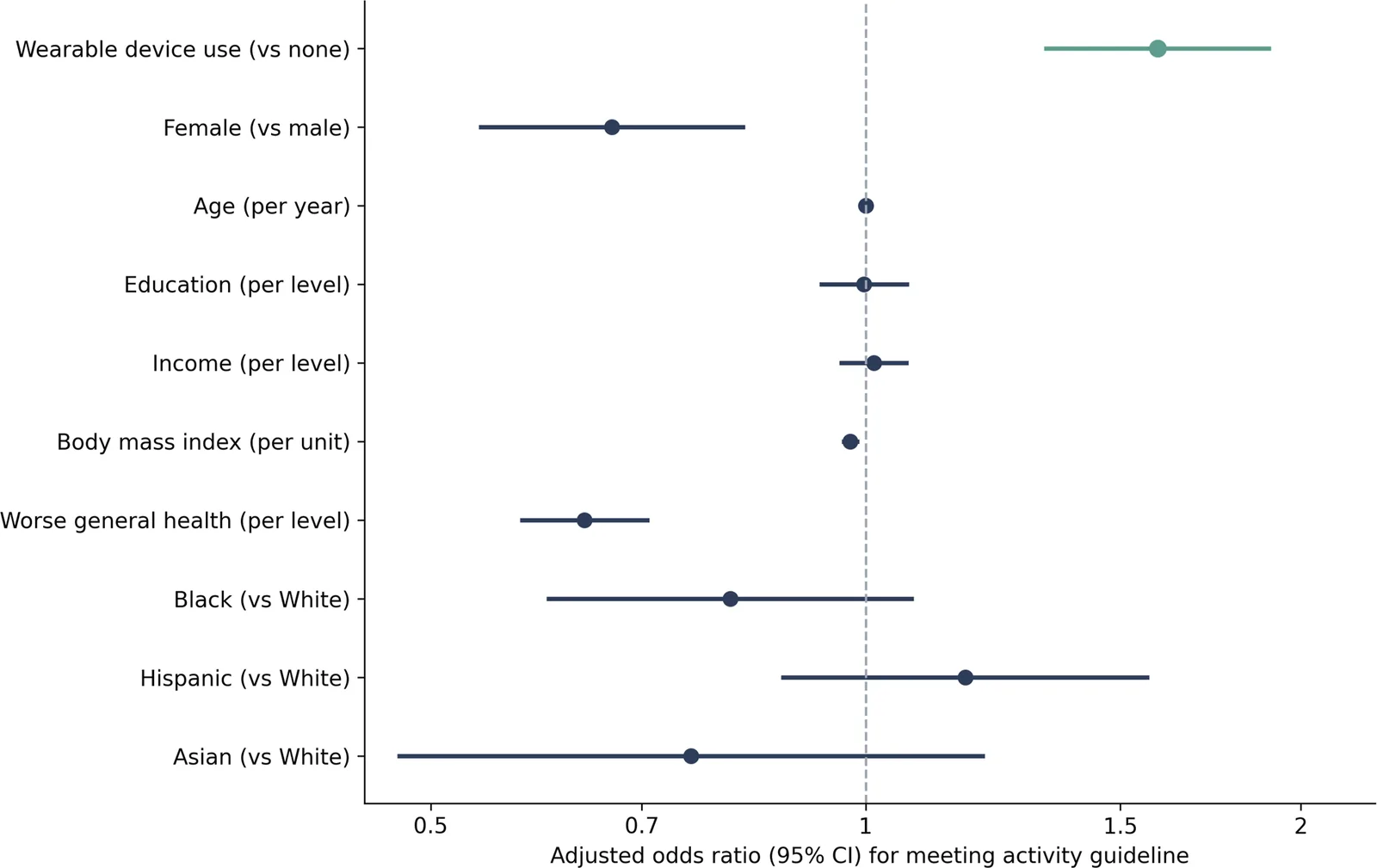
Adjusted odds ratios (95% CI) for meeting the aerobic activity guideline from the weighted multivariable logistic regression (n = 6046). The reference line is at OR = 1.0

### Consistency Across Anxiety Status

The association between wearable use and meeting the guideline was directionally consistent across mental-health strata (Figure 1). Among adults with elevated anxiety symptoms, 48.2% of wearable users met the guideline compared with 28.1% of non-users; among adults without elevated symptoms, the corresponding figures were 48.0% and 38.2%. In fully adjusted stratified models, the wearable OR was 1.75 (95% CI, 0.91-3.36) among distressed adults and 1.54 (95% CI, 1.30-1.83) among non-distressed adults. The multiplicative wearable × anxiety interaction term was not statistically significant (OR, 1.37; 95% CI, 0.73-2.61; *P* = .33). Thus, although the point estimate was numerically larger in the distressed subgroup, the data do not support a statistically distinct benefit. Rather, the data indicate that the positive association between wearable use and guideline adherence is preserved among psychologically distressed adults.

### Secondary Analysis: High-Guideline Threshold (≥300 min/wk)

Weekly activity volume differed by wearable use (Table 1). Compared with non-users, wearable users were less likely to report fewer than 150 min/wk (51.9% vs 63.6%) and more likely to report 150-299 min/wk (27.3% vs 16.5%), whereas the proportion reporting 300 min/wk or more was similar (20.8% vs 19.9%). Accordingly, wearable use was not associated with meeting the high guideline of ≥300 min/wk (unadjusted OR, 1.06; 95% CI, 0.85-1.31; adjusted OR, 1.09; 95% CI, 0.84-1.40; *P* = .52). When adults meeting the high guideline were compared with those not meeting the 150-minute guideline (excluding adults reporting 150-299 min/wk; n = 4762), wearable use was associated with modestly higher odds (adjusted OR, 1.34; 95% CI, 1.04-1.71; *P* = .02). Neither association differed by anxiety status (interaction *P* = .88 and *P* = .62, respectively) (Table 2).

## Discussion

In this nationally representative analysis of US adults, use of a wearable health-tracking device was independently associated with approximately 60% higher odds of meeting aerobic physical activity guidelines, an association that persisted after adjustment for a broad set of sociodemographic and health covariates and that was directionally consistent among adults reporting elevated anxiety symptoms. These findings position wearable devices as a potentially broadly applicable adjunct within the physical-activity pillar of lifestyle medicine.

The magnitude and robustness of the association are consistent with the trial literature. Meta-analyses of wearable and smartphone-based interventions report reliable, if modest, increases in daily activity, attributed to the self-monitoring, feedback, and goal-setting functions that these devices operationalize.^19-22^ Our population-level estimate complements this evidence by demonstrating that, outside the controlled-trial setting, device users are considerably more likely to reach a clinically meaningful activity threshold. That the association was largely unchanged after adjustment for education, income, and general health suggests it is not merely a proxy for socioeconomic advantage or baseline wellness, although residual confounding by unmeasured motivation and health orientation remains plausible. The secondary analyses refine this picture: wearable users were more likely than non-users to reach 150-299 min/wk but were no more likely to exceed 300 min/wk. This pattern suggests that the association is concentrated at reaching the minimum guideline rather than at higher activity volumes, consistent with device goals that typically anchor on daily or weekly targets near the minimum recommendation. Wearables may therefore be most useful for moving insufficiently active adults toward the 150-minute threshold, whereas progression toward the upper range may require additional behavioral support.

The mental-health findings warrant careful interpretation. Adults with anxiety and depression are markedly less active than the general population and face distinctive motivational barriers.^9-13^ We found that wearable users with elevated anxiety symptoms met the guideline at nearly the same rate as users without such symptoms (48.2% vs 48.0%), whereas among non-users, the distressed group lagged well behind (28.1% vs 38.2%). This pattern is compatible with the hypothesis that self-monitoring technology helps distressed adults overcome activity barriers. However, the formal interaction test was not statistically significant, and the stratified estimate in the distressed subgroup, although larger in magnitude, did not reach significance, likely reflecting the smaller subgroup size. We therefore frame the mental-health result conservatively: the association with wearable use is preserved, not clearly amplified, among psychologically distressed adults. Given the established role of physical activity in preventing and reducing anxiety and depressive symptoms,^7,8,11^ even a preserved association is clinically relevant for this high-need group.

These results carry practical implications for lifestyle-medicine clinicians. Recommending or facilitating wearable self-monitoring is a low-intensity, scalable intervention that aligns with established behavior-change theory,^14-17^ and our data suggest its correlates extend to patients with common mental-health symptoms. Because self-monitoring is a transdiagnostic, low-risk technique, it can be integrated into brief clinical encounters and paired with goal-setting and follow-up to reinforce activity gains. At the same time, the sociodemographic gradient in device use (younger, more educated, higher-income, and healthier adults were more likely to be users) signals a digital-health equity concern.^22^ If wearables become a standard behavioral adjunct without deliberate access strategies, they risk widening rather than narrowing activity disparities. Programs that subsidize devices, integrate loaner or reimbursement models into clinical workflows, or embed devices in community settings may be needed to ensure equitable access to benefits. A complementary and lower-cost strategy is to increase awareness of self-monitoring technology that patients already own. Approximately 91% of US adults own a smartphone,^30^ and most smartphones include built-in, accelerometer-based step counting accessible through free or preinstalled apps. In controlled walking tests, smartphone step counts have shown accuracy comparable to that of wearable devices,^31,32^ and interventions using smartphone apps or activity trackers have increased physical activity in pooled trial evidence.^21^ Phones are not always carried, so they can undercount steps in free-living conditions,^33^ but encouraging patients to use the step-counting features of a device they already own may achieve broader uptake than recommending the purchase of a new device, particularly among older and lower-income adults, in whom wearable adoption is lowest.^22^ However, caution may be warranted for those with health-related anxiety, as few commercially available devices have been validated for even one biometric measure,^34^ and inaccurate readings, particularly those that underestimate values, may negatively affect self-esteem and mental health.^35,36^

### Limitations

Several limitations temper these conclusions. First, the cross-sectional design precludes causal inference; more active individuals may be more likely to adopt wearables (reverse causation), and unmeasured factors such as intrinsic motivation may confound the association. Second, physical activity, wearable use, and psychological symptoms were self-reported and subject to recall and social-desirability bias; self-reported activity in particular tends to overestimate true levels. Third, HINTS 7 assesses activity of at least moderate intensity with a single item and does not separately measure vigorous-intensity activity. Vigorous minutes could not be double-weighted as the Guidelines recommend,^3,4^ so adults who meet the guideline mainly through vigorous activity (e.g., 75-149 min/wk of vigorous activity) may have been misclassified as not meeting it, which would underestimate adherence; whether this misclassification differed by wearable use is unknown. Fourth, the anxiety measure was a brief screen rather than a diagnostic assessment, and the threshold used, while conventional, is not diagnostic. Fifth, the wearable item captured use for health tracking during the prior year, but not device type or duration of use. Although HINTS 7 asked users how often they had used a wearable device to track their health in the past month, we did not model frequency of use, so dose-response relationships were not examined. The item also did not capture self-monitoring with smartphone apps alone, so some non-users may have tracked activity with their phones, which would likely bias estimates toward the null. Finally, although we applied the jackknife replicate-weight variance estimator recommended for HINTS public-use data, item non-response reduced the analytic sample for adjusted models.

### Future Directions

Prospective cohort and randomized designs are needed to determine whether wearable adoption causally increases activity and whether effects are larger in psychologically distressed populations, in whom the present study was underpowered to detect effect modification. Trials embedding wearables within lifestyle-medicine programs should measure objective activity and mental-health outcomes, assess sustained adherence, and test equity-focused implementation strategies, including lower-cost smartphone-based self-monitoring, to ensure benefits reach lower-resourced groups. Future work should also validate the accuracy of consumer wearable devices and characterize their psychological effects, particularly among individuals prone to health-related anxiety, for whom inaccurate or underestimated feedback may be counterproductive.

## Conclusions

In a nationally representative sample of US adults, wearable health-tracking device use was independently associated with substantially higher odds of meeting aerobic physical activity guidelines, and this association was preserved among adults with elevated anxiety symptoms. Wearables represent a promising, scalable adjunct for advancing the physical-activity pillar of lifestyle medicine, including among psychologically distressed patients who stand to benefit most. Prospective and interventional studies, with attention to equitable access, are needed to establish causality and optimize deployment.

## Data Availability

The HINTS 7 data that support the findings of this study are publicly available from the US National Cancer Institute (https://hints.cancer.gov). The analysis code is available from the corresponding author on reasonable request.
